# Global Transcriptional Profiling of Longitudinal Clinical Isolates of *Mycobacterium tuberculosis* Exhibiting Rapid Accumulation of Drug Resistance

**DOI:** 10.1371/journal.pone.0054717

**Published:** 2013-01-23

**Authors:** Anirvan Chatterjee, Dhananjaya Saranath, Purva Bhatter, Nerges Mistry

**Affiliations:** Department of Tuberculosis, The Foundation for Medical Research, Mumbai, India; University of Cape Town, South Africa

## Abstract

The identification of multidrug resistant (MDR), extensively and totally drug resistant Mycobacterium *tuberculosis* (*Mtb*), in vulnerable sites such as Mumbai, is a grave threat to the control of tuberculosis. The current study aimed at explaining the rapid expression of MDR in Directly Observed Treatment Short Course (DOTS) compliant patients, represents the first study comparing global transcriptional profiles of 3 pairs of clinical *Mtb* isolates, collected longitudinally at initiation and completion of DOTS. While the isolates were drug susceptible (DS) at onset and MDR at completion of DOTS, they exhibited identical DNA fingerprints at both points of collection. The whole genome transcriptional analysis was performed using total RNA from H37Rv and 3 locally predominant spoligotypes viz. MANU1, CAS and Beijing, hybridized on MTBv3 (BuG@S) microarray, and yielded 36, 98 and 45 differentially expressed genes respectively. Genes encoding transcription factors (*sig*, *rpoB*), cell wall biosynthesis (*emb* genes), protein synthesis (*rpl*) and additional central metabolic pathways (*ppdK, pknH, pfkB)* were found to be down regulated in the MDR isolates as compared to the DS isolate of the same genotype. Up regulation of drug efflux pumps, ABC transporters, trans-membrane proteins and stress response transcriptional factors (*whiB*) in the MDR isolates was observed. The data indicated that *Mtb*, without specific mutations in drug target genes may persist in the host due to additional mechanisms like drug efflux pumps and lowered rate of metabolism. Furthermore this population of *Mtb*, which also showed reduced DNA repair activity, would result in selection and stabilization of spontaneous mutations in drug target genes, causing selection of a MDR strain in the presence of drug pressures. Efflux pump such as *drrA* may play a significant role in increasing fitness of low level drug resistant cells and assist in survival of *Mtb* till acquisition of drug resistant mutations with least fitness cost.

## Introduction

Globally *Mycobacterium tuberculosis* (*Mtb*) results in 8 million new cases of tuberculosis (TB) and about 2 million deaths annually [1]. In India, TB infection is a grave public health problem with an annual incidence of 185/100,000 [2]. Mumbai, in the western region of India is a highly populated metropolis, with an annual TB incidence of 299/100000 [3]. The emergence of multidrug resistance (MDR) and extensively drug resistance (XDR) along with the recent reports of totally drug resistant tuberculosis (TDR) [4] poses a threat to the TB control programme patterned on Directly Observed Therapy Short Course (DOTS) strategy. Our study in DOTS compliant patients showed that 32% of drug susceptible (DS) isolates at onset, developed MDR at the end of 5th month of therapy without exhibiting a change in the DNA fingerprint (DF) as determined by spoligotyping and 6 loci MIRU-VNTR (Unpublished data). A subset of these patients was investigated for the current study. While complete genomic identity of the isolates may be best ascertained by whole genome sequencing, it was not feasible in the current study.

While the molecular basis of drug resistant *Mtb* has been attributed to point mutations in identified drug target genes [5], the mechanisms for rapid accumulation of drug resistance involving multiple gene loci are yet to be elucidated. The risk of mutation per bacterium per cell division for each of the anti-mycobacterial drug has been estimated earlier to be 3.32×10^−9^ for rifampin, 2.56×10^−8^ for isoniazid and 1.0×10^−7^ for ethambutol [6]. Further simultaneous mutation to more than one drug would be a multiplicative probability of mutation rates of each of the drug target genes [5]. Thus the mutation rate for rifampicin, isoniazid and ethambutol would be 8.5×10^−24^. A minimal probability of 10^−6^ per gene and 10^−18^ for the three drug resistance genes makes the possibility of multiple drug resistance in *Mtb* during the DOTS treatment rare, and is indicative of alternative mechanisms for acquisition of DR. Besides, alternative drug resistance mechanisms associated with efflux pumps [7], [8], [9], [10], [11], transporter proteins [12] and DNA mismatch repair proteins [13], [14], have been reported to provide low level resistance to multiple antibiotics. The resistance conferred by such mechanisms may act through switching on/off genes, as well as through differential expression of genes in the presence of drugs or other stress factors [15].

To understand the mechanisms of rapid accumulation of drug resistance in *Mtb*, the current study was designed to detect changes in global transcriptional levels of genes in 3 pairs of longitudinal clinical *Mtb* isolates with identical DF, from drug compliant patients showing amplified drug resistance. The metropolis of Mumbai in western India, with a high incidence of TB and high proportion of MDRTB [3], [4] provided an opportunity to study rapid evolution of MDR in a region of high disease prevalence with simultaneous presence of varied selective pressures including heterogeneous human demography, drug pressures and immuno-compromised hosts [16].

Hence we examined transcriptional profiles of 3 predominant spoligotypes, MANU1, CAS and Beijing, isolated from patients in the region [17]. While MANU1 was the most predominant spoligotype in the region [17], [18], CAS is extensively found in north India [19]. The Beijing strain found globally [20] has been known to cause micro epidemics [21].

Whole genome expression analysis using a pan genome array design encompassing 4274 open reading frames (ORF) derived from 8 published TB complex genomes of H37Rv, CDC1551, AF2122/97, H37Ra, F11, KZN1435, BCG Pasteur and BCG Tokyo was employed. The arrays were procured from Bacterial Microarray Group, St George’s, University of London, London, U.K.

## Methods

### Mtb Isolates

The *Mtb* isolates were collected from the patients enrolled in ‘The Revised National Tuberculosis Control Program (RNTCP)’, in four municipal wards in Mumbai city. This study was part of a larger epidemiological project studying transmission of MDRTB in an endemic setting in these wards, covering a population of 3 million residents comprising 38 DOTS Centers.

### Patients

During the period April 2004 to September 2007, sputum samples were collected from newly diagnosed, smear positive, previously untreated pulmonary tuberculosis patients, registered with RNTCP at 2 longitudinal time points viz pretreatment *Mtb* isolate and a 5^th^ month follow-up sample. Patients with a history of TB or previous antitubercular therapy, as determined through a questionnaire and scrutiny of district TB registers, were excluded. Follow-up samples were collected only from patients who had not defaulted and had undergone uninterrupted DOTS therapy. The isolates were tested for drug susceptibility by a previously described radio-respirometric assay [3]. The isolates were subjected to DNA fingerprinting using spoligotyping and 6 loci MIRU-VNTR techniques [22], [23]. Sixteen isolate pairs with identical DF, were identified as drug susceptible (DS) at onset, and were found to be MDR at 5^th^ month post treatment. Further, global transcriptional profiles of 3 longitudinal pairs of clinical isolates of spoligotype MANU1, CAS and Beijing, were investigated. Although sample size was limited, previous studies have performed global transcriptional profiling (GTP) of limited clinical samples (3 patient isolates) [1] or with laboratory *Mtb* strains like H37Rv, H37Ra [24], [25], [26], [27], [28].

### Ethics Statement

The study was approved by the Foundation for Medical Research (FMR) Institutional Ethics Committee (20.07.2001/01). Patients were recruited only after they provided written informed consent.

### Bacterial Cultures

The sputum samples were inoculated on Lowenstein Jensen (LJ) medium from the longitudinal samples of patients at onset of DOTS and 5^th^ month post treatment. Briefly, the sputum samples were collected in CPC-NaCl vials and concentrated by Petroff’s method [3]. The concentrated samples were inoculated onto single LJ slants (Hi-Media, Delhi, India) and incubated at 37°C until growth was observed. A single longitudinal isolate pair of each spoligotype (MANU1, CAS, Beijing) and drug susceptible *Mtb* strain H37RV, were used for RNA extraction and whole genome transcriptional analysis.

### RNA Extraction

Total RNA was extracted from the mycobacterial cultures as per the method described by Mangan et.al.,1997 [29]. Briefly, bacterial cultures were washed twice with Tris EDTA (TE) buffer (pH8.0), treated with 1 ml of RNA stabilizing agent from Rneasy mini kit (Qiagen,Valencia, USA) for 5 min, followed by washing in TE buffer and suspended in 20 µl TE buffer. Zirconium beads in a 1∶1 proportion to the bacterial volume were added along with lysozyme (10 g/l) and placed in an orbital shaker for 60 min. Total RNA was extracted using 1 ml TRIzol (Life technologies, USA) with constant shaking on a vortex mixer for 15 min. The solution was incubated at 15–30°C with 0.2 ml chloroform per ml of TRIzol for 5 min, and centrifuged at 12000 g for 10 min at 4°C. The RNA in the aqueous phase was recovered by addition of 0.5 ml isopropyl alcohol, incubated at 15–30°C for 10 min, and precipitated by centrifugation at 12000 g for 15 min. The pellet was washed in 1 ml 75% ethanol, dried and resuspended in 25 µl RNase free water (Qiagen, California, USA).

RNA concentration and purity were determined on Nanodrop (Thermo-Fisher, USA). RNA integrity number (RIN) was ascertained using Agilent 2100 Bioanalyzer (Agilent Technologies Inc, USA). The RNA with RIN>8.5 was used for microarray hybridization.

### Microarray Slide Design

An Agilent 8×15 K, 60 mer oligonucleotide array printed on a glass microscope slide was procured from Bacterial Microarray Group, St George’s, University of London. The microarray was constructed, according to the method described in McCarthy 2011 [30]. A pan-genomic redundant set of genes representing *Mtb* strains H37Rv (Ensembl Bacteria Release 2 (http://bacteria.ensembl.org/(EB)), CDC1551 (EB), H37Ra (EB), F11 (NC_009565; NCBI Entrez), KZN 1435 (NC_012943; NCBI Entrez), and *M. bovis* strains AF2122/97 (EB), BCG (EB), BCG Tokyo 172 (NC_012207; NCBI Entrez) was used to design the probes. Briefly, all unique genes from the 4000 chromosomal predicted coding sequences of *Mtb* strain H37Rv were initially selected. A subsequent iterative process was used to determine genes absent from H37Rv or with significant divergence based on BLASTN bit scores. For each of the determined redundant gene set, multiple optimal hybridisation 60-mer oligonucleotide sequences were designed (Oxford Gene Technologies), from which a minimal non-redundant subset of oligonucleotides were selected with target coverage of three 60-mers per gene, resulting in a set of 12824 oligos. Arrays were manufactured on the Inkjet in-situ synthesized platform (Agilent) using the 8×15 k format. The complete array design is available in BµG@Sbase (BµG@Sbase: A-BUGS-41) and also in ArrayExpress (ArrayExpress: A-BUGS-41).

Microarray hybridisation was performed at Agilent facility available at “iLifediscoveries ltd”, Global Discovery Center - India, Gurgaon, India. A total of 11 µg (1 µg/µl) RNA was hybridized as per Two-Color Microarray-Based Prokaryote Analysis FairPlay III Labeling protocol stated by the manufacturer (Agilent Technologies ltd., USA). Each array of the *MTB*v3 slide was hybridized with RNA extracts from two isolates labeled with either Cy3 or Cy5 dyes. Additionally to reduce error rates, we performed dye swap, wherein the labels were reversed for each hybridization. The 8 arrays were hybridized as follows: Array 1 & 8– H37Rv labeled with Cy3 and Cy5; Array 2– MANU DS labeled with Cy3 and MANU MDR labeled with Cy5; Array 3– MANU DS labeled with Cy5 and MANU MDR labeled with Cy3; Array 4– Beijing DS labeled with Cy3 and Beijing MDR labeled with Cy5; Array 5– Beijing DS labeled with Cy5 and Beijing MDR labeled with Cy3; Array 6– CAS DS labeled with Cy3 and CAS MDR labeled with Cy5; Array 7– CAS DS labeled with Cy5 and CAS MDR labeled with Cy3.

The design of the microarray represented 4000 genes in triplicates constituting 12824 oilgo probes from multiple genomes with minimal cross hybridisation. Besides, Cy3/Cy5 dye swap of all hybridisations and normalization of all signals ensured minimal error rates. Further t-test with Benjamini and Hochberg’s multiple testing correction [31], minimised false positives.

### Data Analysis

The microarray slide was scanned and the gene expression data sets were extracted into a Cel file (raw intensity file). The data was analyzed using GeneSpring 11.5 software, followed by differential gene expression and clustering analysis. Fully annotated microarray data have been deposited in BµG@Sbase (accession number E-BUGS-134; http://bugs.sgul.ac.uk/E-BUGS-134) and also ArrayExpress (accession number E-BUGS-134).

#### Data preprocessing and normalization

The initial microarray data (txt) for control and test sets were preprocessed using Robust Multichip Average (RMA) algorithm consisting of three steps: a background adjustment, quantile normalization and summarization.

#### Differential gene expression analysis

Differential gene expression analyses were performed using pair wise comparison for each experimental set. Further, t-test with Benjamini and Hochberg’s multiple testing correction [31] was performed, minimising false positives. A p<0.05 was considered as significant differential expression. While a p<0.05 has been used as a cut off to determine differential expression in global transcriptional profiling of *Mtb* [26], [32], we have also investigated a subset of genes with a more stringent cut off of p<0.005. The statistical test above generated a test value or a statistic called the test metric for each gene with a larger test metric indicating larger differential expression. Under the assumption that the expression values for a gene within each group were normally distributed and the variances of the normal distributions associated with the two groups were same, the above computed test-metrics for each gene were converted into p-values, in most cases using closed form expressions.

#### Fold Change (FC) calculation

Fold change was calculated between a drug sensitive and drug resistant isolates of the same strain. Additionally fold change differences were also analysed between H37Rv and the clinical DS isolates of MANU, CAS and Beijing. Thus quantitative differential expression was indicated by the ratio of normalized intensities between the average intensities of the grouped samples. Genes which yielded a differential fold change ≥2 were included in further analysis, and a profile plot was generated for differentially expressed genes.

#### Hierarchical Clustering (Hcl)

Hierarchical clustering was performed using an agglomerative approach. There are several important parameters, which control the order of merging entities and sub clusters in the dendrogram. In the current study Hcl was performed on “conditions and genes”. The average linkage method and Euclidean distance metric was selected. The Hcl expression image is represented as a dendrogram, an intuitive view of the results of the clustering method. The expression image tree further characterizes on the basis of color wherein the red, blue and yellow colors represent over expression, under expression and equal levels of expression respectively.

## Results

### Whole Genome Microarray Based Expression Analysis

#### i. Differentially expressed genes in MDR clinical isolates as compared to DS clinical isolate with identical DF

**Figure 1:**
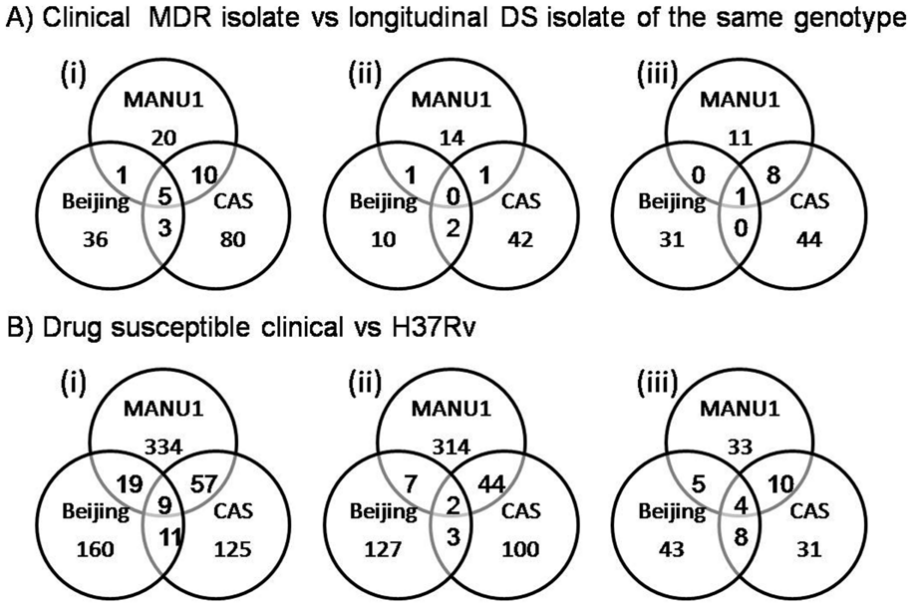
Number of genes differentially regulated (p≤0.05) between DS clicinal isolates as compared to respective MDR clinical isolate. (i) Total number of genes differentially regulated; (ii): Up regulated genes; (iii): Down regulated genes. The numbers in the overlap regions indicate the common genes differentially expressed.

A differential expression (p<0.05) of genes was observed between the MDR and DS isolates of MANU1, CAS and Beijing as indicated in Figure 1a. Thirty six genes were found to be differentially regulated in the MANU1 MDR isolate as compared to the pre-treatment DS MANU1 isolate from the same patient; 98 genes were differentially expressed in the CAS MDR isolate as compared to the CAS DS isolate; and the Beijing MDR isolate showed 45 genes differentially expressed with respect to the DS Beijing isolate. Of the differentially regulated genes, 44%, 46% and 29% were up regulated in the MANU1, CAS and Beijing MDR isolates respectively, as compared to DS isolate with identical DF. Upregulation of genes in MDR isolates was between 2 to 26.2 fold, and down regulation of genes was between 2 to 23.7. The fold changes have been further detailed in the individual genes in Table 1.

**Table 1:** Functional categories of differentially expressed genes (p<0.05) between longitudinal clinical isolates of the same strain.

Function	MANU1		CAS		Beijing	
	Up (X fold)	Down (X fold)	Up (X fold)	Down (X fold)	Up (X fold)	Down (X fold)
CellMetabolism	Rv3093c (2),Rv1144 (2),Rv3715 (2)	Rv2770c (2), Rv2968c (2), Rv0400c (2), Rv0456c (2.5), Rv3177 (3.5), Rv2934 (2),Rv3793 (4)	Rv2094c (3),Rv1884c (4),Rv2427c (2),Rv2934 (5.5),Rv3290c (4), Rv1925 (2),Rv3854c (3.5), Rv2792c (5),Rv3161c (4), Rv0677c (2),ACTB (3.5), Rv3217c (2)	Rv2450c (2), Rv2029c (2),Rv0555 (2.5), Rv2988c (3),Rv3842c (3), Rv2150c (2),Rv2007c (20), Rv1467c (2),Rv2199c (3), Rv1589 (2),Rv3793 (9), Rv1735c (3),Rv2345 (3), Rv3882c (3)	Rv0568 (2), Rv2094c (4), Rv2429 (2)	Rv0187 (2), Rv1127c (2), Rv1328 (2), Rv1712 (4), Rv3464 (4), Rv3535c (3), Rv3607c (2), Rv1140 (3), Rv1476 (4), Rv2518c (3)
Transcription, translation, replication	Rv0830 (2),Rv1788 (2),TBFG_11219 (3), Rv0667 (3),Rv3911 (3),Rv3219 (14)	Rv0005 (3),Rv0006 (4)	Rv3219 (11), Rv1994c (26),Rv0302 (2), Rv2745c (3),Rv3583c (3), Rv0006 (4)	Rv3143 (14), Rv1221 (12),Rv0667 (5), Rv0991c (2),Rv3879c (4), Rv1983 (2),Rv1630 (3), Rv0706 (3),Rv0723 (4), Rv0702 (2)	Rv0767c (2), Rv1221 (9), Rv3414c (14), Rv0005 (4), Rv2442c (2), Rv2839c (4.5)	Rv0735 (15), Rv2728c (8), Rv3911 (16), Rv0058 (3), Rv1266c (4), Rv2357c (7), Rv2904c (3), Rv3919c (3)
Drug resistance mechanisms (Efflux pumps, Multi-drug resistantproteins)	Rv3065 (4),Rv2936 (7),Rv1687c (7)	Rv1686c (4),Rv2937 (4),Rv2025c (5.5),Rv1457c (7.5)		Rv2846c (8), Rv2937 (9),Rv2936 (6), Rv1458c (3),Rv1634 (7.5), Rv1686c (3),Rv2025c (7), Rv3065 (9)	Rv1686c (4.5), Rv2936 (4), Rv2937 (2)	Rv0783c (6)
DNA repair		Rv3014c (6),Rv2737c (6),Rv1633 (6),Rv0630c (7)	Rv2736c (2),Rv2720 (5)	Rv1633 (7), Rv1638 (12.5),Rv3715c (8), Rv0630c (7),Rv3014c (12), Rv0005 (15)		Rv0003 (15), Rv0630c (24), Rv2592c (5)
ImmuneFunction	Rv3881c (3)		TBMG_00064 (2),Rv3891c (2.5)	Rv2031c (6)		
Unknown	Rv0756c (2), Rv2137c (2), Rv1871c (3)	Rv2619c (2),Rv2184c (2),Rv2082 (2)	SM_11 (2), Rv2166c (5),Rv1593c (4), Rv0108c (6),Rv0282 (2), Rv0502 (3),Rv0515 (5), Rv0626 (2),Rv0863 (2), Rv1148c (2),Rv1540 (2), Rv1883c (4),Rv1945 (5.5), Rv2016 (4),Rv2255c (3), Rv2472 (2),Rv2714 (3.5), Rv2777c (2),Rv2827c (6), Rv2954c (3),Rv3188 (3), Rv3371 (2),Rv3642c (3)	Rv2623 (3), MT2466 (8),MT1356 (3), Rv0049 (2),Rv0569 (5), Rv1738 (9),Rv1813c (9), Rv1841c (2),Rv2030c (4.5), Rv2596 (5),Rv2626c (10.5), Rv2694c (9),Rv3615c (2), Rv3831 (2)	Rv0626 (2)	MT1040.1 (5), Rv0141c (4), Rv0340 (3), Rv0875c (4), Rv0876c (2), Rv1766 (5), Rv2310 (3), Rv3088 (4), Rv3698 (2), Rv3701c (2)

1. “Up” and “Down” denote regulation in MDR isolate w.r.t. DS isolate.

2. Numerical within parenthesis indicate fold change.

3. FC range.

a. MANU1: 2 to 13.6 in up regulated genes, and 2 to 7.5 in down regulated gene.

b. CAS: 2 to 26.2 in up regulated genes, and 2 to 20 in down regulated gene.

c. Beijing: 2 to 13.7 in up regulated genes, and 2 to 23.7 in down regulated gene.

**Figure 2:**
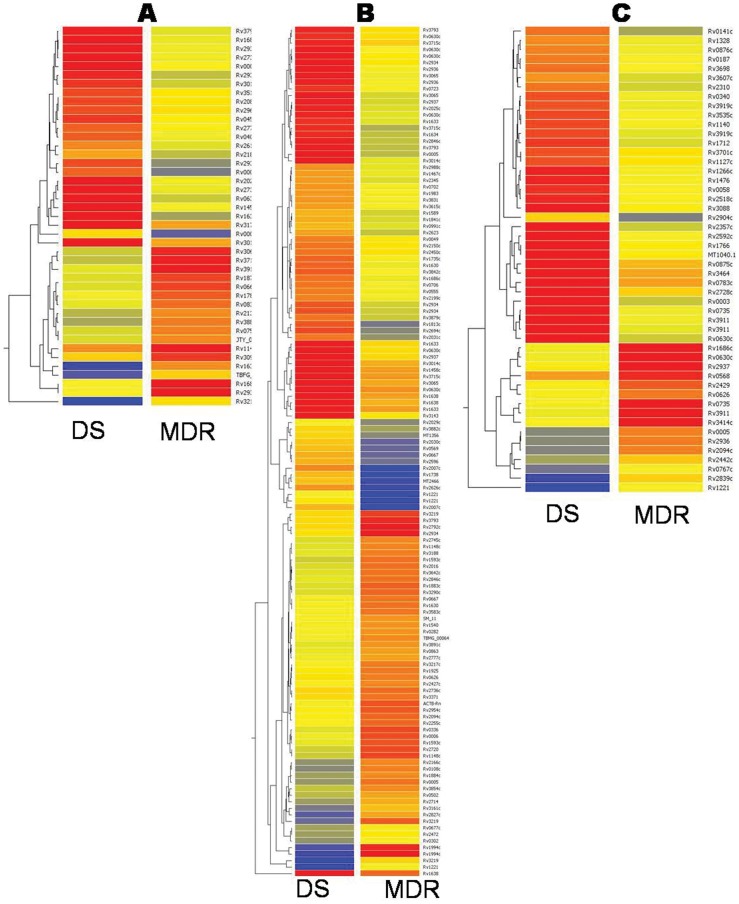
Hierarchical clustering. Hierarchical clustering of genes in (A) MANU1; (B) CAS (C) Beijing.

The differentially expressed genes in the longitudinal isolate pairs were broadly classified into 5 functional categories with an additional group for unknown functions (Table 1). Various functional gene families differentially regulated in the MDR strain as compared to the respective DS strain were: cell metabolism (*emb, pfk, ppd* gene families); DNA metabolism (*sig, rpo, whiB*,*rps, rpl* gene families); drug resistance mechanisms including efflux pumps (*mmr, drr, efp* gene families); DNA repair (*rec, ruv* gene families) and genes associated with immune function (*hsp* gene family, and *Mtb* antigens). The *gyr* gene which is the only type II topoisomerase encoded by the *Mtb* genome [33] and the drug target of fluoroqinolones (FQ), was also up regulated in the MDR isolates. The clustering data analysis of the differentially expressed genes is represented as a hierarchical cluster plot for each of the three genotypes (Figure 2). *rpoβ*, the rifampicin drug target gene, was down regulated in MANU1 MDR, down regulated in MDR CAS and showed no significant deregulation in MDR Beijing isolate. The *gyrB* gene, associated with flouroquinolone resistance was down regulated in MANU1 and CAS MDR isolates, but up regulated in Beijing MDR isolate. The *embC* gene, showed down regulation in both MDR MANU1 and MDR CAS. The *embB, katG* and *inhA* showed no significant deregulation in any of the MDR isolates. Detailed information including the differential fold change, gene symbol, accession number, regulation and functional category is given in Table S1.

Conventionally microarray analysis of differentially expressed genes in *Mtb* transcriptomics, have been analysed at p<0.05 threshold [32], as reported by Makarov and colleagues using H37Rv cultured in the presence and absence of drugs [26]. Hence, following precedent and to enable comparison with international data, our initial analysis focused on differential expression at p<0.05. While the genes analysed at p<0.05, were indicative of various process associated in rapid amplification of drug resistance, the study also has also investigated a subset of genes with (p<0.005) (Table 2). These genes represent candidate genes for further studies to establish their role in drug resistance amplification. As compared to DS MANU1, the MDR MANU1 showed upregulation of *rpoβ* and *sigM*, which are part of transcriptional mechanism, along with upregulation of efflux pump Rv1687c. Conversely, the MANU1 MDR isolate also showed down regulation of DNA repair genes and efflux pumps. The CAS MDR isolate showed upregulation of various genes responsible for translation, and down regulation of efflux pumps and DNA recombination, as compared to the DS CAS isolate. While the MDR Beijing strain did not show any upregulated genes as compared to the DS Beijing isolate, it showed down regulation of genes involved in replication, transcription and translation (Table 2).

**Table 2:** Genes with significant differential expression (p<0.005).

Strain pair	Up regulated genes	Down regulated genes
MDR MANU1 vs DS MANU1	*rpoβ, sigM*, 1687c	*embC*,Rv1686c, *drrB, uvrB, recB, recA,echA2,* Rv1457c
MDR CAS vs DS CAS	*tatA, mmpS5, ppsD, lexA, whiB1*, Rv0282	*rplV, sigE, rpsA, efpA, pfkB, uvrB,* Rv3615c, Rv0706, Rv3879c, Rv0569, Rv1221, Rv1630, Rv2846c, Rv3831, Rv2029c, Rv2025c
MDR Beijing vs DS Beijing		*sigL, rpsL, recF*, Rv2728, Rv3701

#### ii. Comparative analysis of differentially expressed genes in the DS clinical isolates as compared to H37Rv

A total of 715 genes were differentially expressed at a statistically significant level (p<0.05) in the three DS clinical isolates in comparison to H37Rv. MANU1, CAS and Beijing isolates showed 419, 202 and 199 differentially expressed genes respectively, as compared to H37Rv (Figure 1b). Of these 70–88% genes were up regulated, while 12–30% were down regulated (Figure 1b). The differentially expressed genes in the DS clinical isolates were functionally associated with cell metabolism, transcription, translation, replication, DNA repair, immune function and efflux pumps. The over expression of genes ranged from 2–13.6 fold in MANU1 DS, 2–30.6 fold in CAS DS, and 2–16.4 fold in Beijing DS isolates as compared to H37Rv. The fold change in down regulated genes of MANU1, CAS and Beijing DS isolates as compared to H37Rv was 2–8.3, 2–13.6 and 2–30 fold respectively. Nine genes were differentially expressed in all the three isolates, of which 2 genes were up regulated and 4 genes were down regulated in the three strains. Of the remaining 3 genes, one was up regulated in the CAS and MANU1 isolates but down regulated in the Beijing isolate. The remaining 2 genes were down regulated in the CAS and Beijing isolates but up regulated in the MANU1 isolate. However one non-annotated gene Rv3407, which is being studied as a vaccine candidate [34] was down regulated in all 3 DS isolates when compared to H37Rv.

The details of the genes with fold changes, p values, gene symbol, accession number and functionality are listed in Table S2.

## Discussion

The focus of the study was to understand mechanisms of rapid amplification of drug resistance by identification of differentially expressed genes in longitudinal clinical isolates. These pairs of isolates were DS at diagnosis and exhibited MDR after five months of compliant DOTS treatment, and had identical DF as determined by spoligotyping and 6 loci MIRU-VNTR. In the current study, it was not feasible to confirm the complete genomic identity of the isolates by whole genome sequencing. In our earlier study, we observed 247 *Mtb* isolates from the same population, showed a high discriminatory index of 0.98 on fingerprinting by spoligotyping and MIRU-VNTR (Unpublished data), indicating suitability of using the fingerprint data to ascertain strain identity. The presence of a high proportion of MDR in a DOTS prescribed study population provided the opportunity to detect effects of drug pressures on global gene expression in clinical isolates of *Mtb*. We studied three such longitudinal isolate pairs representing the locale predominant spoligotypes: MANU1, CAS and Beijing [17], [18].

Of the 3 strains, the CAS MDR isolate showed highest and the Beijing MDR the lowest number of differentially expressed genes as compared to their respective DS isolate. Various transcription factors (*sig* gene family, *rpoB*), cell wall biosynthesis genes (*emb* gene familly), protein synthesis genes (*rpl*) and several central metabolic pathway genes (*ppdK, pknH, pfkB)* were down regulated in the MDR isolates, indicating an overall reduction in metabolism. The reduction in metabolism provided drug tolerance and persistence of *Mtb in vivo*, since conventional anti-TB drugs target biosynthetic processes involved in cell growth, including transcription, translation and cell wall biogenesis [35]. We also observed various DNA repair and DNA stability genes including *rec*, *uvr, ruv* and *lig* genes down regulated. On the other hand, up regulation of various drug efflux pumps, ABC transporters and trans-membrane proteins (some yet to be annotated) was observed in the MDR isolates, indicative of cellular mechanisms which may impart broad spectrum antibiotic resistance. Up regulation of transcriptional genes associated with antimicrobial stress such as *whiB* gene familly [36], was observed in the MDR isolates.

The selection of mutations in identified drug target genes, *rpoB, inhA, katG, embB* and *gyrA* is gradual and sequential and elicits high fitness costs. Thus the acquisition of these mutations in the presence of a multidrug therapy requires ancillary mechanisms, as indicated in our study. For instance, the *gyrA* gene has been associated with FQ resistance. These mutations have been reported to have high fitness costs [5]. Alternatively efflux pumps have been shown to cause FQ resistance [37]. The up regulation of *gyr* genes in MDR isolates may therefore indicate compensation for FQ resistance which may be due to up regulation of efflux pumps. These observations highlight a need for drug susceptibility testing of second line drugs even in patients undertaking DOTS.

Efflux pumps have been shown to confer low level resistance to various compounds and anti-TB drugs [38]. Additionally slower cellular metabolism and transcription have been associated with drug tolerance in cells without specific drug resistance mutations [35]. Thus from our observations, we infer that even in drug compliant patients, over expression of efflux pumps and under expression of metabolic processes may be responsible for low level resistance to multiple drugs without the presence of multiple drug resistance mutations. Such strains may also exhibit a mutator phenotype [39] due to reduced DNA repair activity observed in the current study. Further exposure of these isolates to anti-TB drugs may cause “*selection and stabilization of spontaneous mutations*”, resulting in clonal expansion of MDR isolates [38], While such mechanisms have been advocated for isoniazid resistance [38], our observations extend these implications to all the first line anti-TB drugs.

Alternate mechanism of rapid amplification of resistance has been hypothesised earlier [40], however it has not been demonstrated in clinical isolates. The three different *Mtb* strains: MANU1, CAS and Beijing, used in our study, have different evolutionary history, inducing different mechanisms of drug resistance. The MANU1 genotype is considered endemic in the area [17], [18] and has been found to be associated with drug susceptibility [17]. Additionally, MDR MANU1 strains have been shown to outcompete drug susceptible CAS and Beijing strains *in vitro* [41]. The CAS strains found predominantly in North India [42] has been shown to induce greater cavitation [17] and consequently result in a more latent disease [41]. The Beijing strains are not only associated with MDR, they were also found to produce most fit MDR strains [41]. Additionally, the Beijing strains have been shown to able to accumulate MDR mutations when exposed to a different host [43]. Thus evidence emphasizes that varied mechanisms may be responsible for drug resistance in these three strains, indicating variations in the gene expression, as seen in our results. Consequently the study may reflect the broad mechanisms of rapid amplification of drug resistance.

Furthermore the observation of upregulation and down regulation of genes involved in the same cellular function including transcription, drug efflux and protein synthesis is indicative of compensation for drug resistance mechanism. For instance the upregulation of Rv1687c, an ABC transporter also known as a drug efflux pump [38], may result in fitness cost due to reduced intake of nutrients. However the down regulation of Rv1686c, a different ABC transporter, may compensate by enhancing selective intake of nutrients. Compensation may also demonstrate the upregulation of *sigE* and *sigM*, genes associated with transcription, along with down regulation of *whiB* gene, which have been known to play important role in transcriptional regulation [44]. Thus compensation (empirical and/or genetic), which results in adaptive evolution leading to drug resistance [45], may have significant role in maintenance of low level drug resistance.

Apart from studying the differential expression of strains with amplified drug resistance, we also examined the differential expression of clinical DS isolates as compared to laboratory standard DS strain H37Rv, initially isolated from a patient in 1905 in the pre-antibiotic era, and which is considered as the neotype of *Mtb* [46]. The comparison of DS clinical isolates and H37Rv provides clues for maintenance and increase of pathogenicity of a clinical strain in the presence of drug and immune pressures. This was evinced by the large number of genes differentially expressed in the clinical DS isolate as compare to H37Rv. While these genes were also up regulated in the DS strain with respect to the respective MDR isolate, their over expression when compared to H37Rv is indicative of a human pathogen under drug pressures, which has accumulated functional mutations with least fitness costs enabling it to infect, survive and transmit within human hosts more efficiently.

The observations from the study provide significant candidate genes for studying novel mechanisms of drug resistance, drug tolerance and persistence of *Mtb*. Efflux pump such as *drrA* may play a significant role in increasing fitness of low level drug resistant cells and assist in survival of *Mtb* till acquisition of drug resistant mutations with least fitness costs. Genes like *sigD, sigM, gyrA, whiB* represent candidates for developing novel markers which predispose *Mtb* strains to drug resistance.

### Limitations of the Study

The small number of clinical isolates (3 pairs) used for GTP, limited by the cost of microarray slides, and scarcity of RNA from clinical samples (reported in earlier studies [1]), was a limitation of the study. However while most previous studies performed GTP with laboratory *Mtb* strains like H37Rv, H37Ra [24], [25], [26], [27], [28], we performed GTP with 3 pairs (6 isolates) of clinical isolates and H37Rv, which is comparable to earlier reports (1–3 patient isolates) [1]. The lack of quantitative validation of the genes is another limitation of the study. Thus the forward path of quantitative real-time PCR on specific genes in relevant biological pathways in increased number of clinical drug susceptible and resistant samples is planned.

## Supporting Information

Table S1 **List of genes differentially expressed between longitudinally collected clinical isolates of the same genotype.**
(XLS)

Table S2 **List of genes differentially expressed between all clinical isolates and H37Rv.**
(XLS)
